# Synaptic proteins and receptors defects in autism spectrum disorders

**DOI:** 10.3389/fncel.2014.00276

**Published:** 2014-09-11

**Authors:** Jianling Chen, Shunying Yu, Yingmei Fu, Xiaohong Li

**Affiliations:** ^1^Shanghai Key Laboratory of Psychotic Disorders, Shanghai Mental Health Center, Shanghai Jiao Tong University School of MedicineShanghai, China; ^2^Department of Neurochemistry, New York State Institute for Basic Research in Developmental DisabilitiesStaten Island, NY USA

**Keywords:** autism spectrum disorders, synaptic protein, GABA, PSD-95, SHANK3, TAOK2

## Abstract

Recent studies have found that hundreds of genetic variants, including common and rare variants, rare and *de novo* mutations, and common polymorphisms contribute to the occurrence of autism spectrum disorders (ASDs). The mutations in a number of genes such as neurexin, neuroligin, postsynaptic density protein 95, SH3, and multiple ankyrin repeat domains 3 (*SHANK3*), synapsin, gephyrin, cadherin, and protocadherin, thousand-and-one-amino acid 2 kinase, and contactin, have been shown to play important roles in the development and function of synapses. In addition, synaptic receptors, such as gamma-aminobutyric acid receptors and glutamate receptors, have also been associated with ASDs. This review will primarily focus on the defects of synaptic proteins and receptors associated with ASDs and their roles in the pathogenesis of ASDs via synaptic pathways.

## INTRODUCTION

Autism spectrum disorders (ASDs) are a heterogeneous group of neurodevelopmental disorders characterized by social communication deficits and stereotyped behaviors with restricted interests (American Psychiatry Association [APA], 2013). Autism was first reported by Kanner (1943), who described seven boys and four girls who exhibited “extreme aloneness from the very beginning of life, not responding to anything that comes to them from the outside world.” Asperger (1944) described four boys with social communication difficulties. During the past 70 years, the definition of autism has developed as understanding of the disorder increased. It was first introduced as infantile autism in the official diagnostic nomenclature in the third edition of the Diagnostic and Statistical Manual of Mental Disorders (DSM-III), was referred to as Pervasive Developmental Disorders (PDD) in DSM-IV and was defined as ASDs in the latest revision of the DSM, DSM-V (American Psychiatry Association [APA], 2013), which was published in May 2013. In DSM-V, ASDs includes disorders that were previously diagnosed separately, such as autistic disorder, Asperger’s disorder, childhood disintegrative disorder, and pervasive developmental disorder not otherwise specified. The decision to merge the three disorders was taken because they could not be easily distinguished from each other. Studies have shown that more than half of adults with autism have poor or very poor outcomes (Howlin et al., 2004; Billstedt et al., 2005) in terms of independent living, educational attainment, employment, and peer relationships.

Autism was considered as a rare childhood disorder, and in the first epidemiological study conducted in the UK in 1966, Lotter (1966) reported a prevalence rate of autism of 4.5 in 10,000 children. However, the prevalence of ASDs has steadily increased in the past two decades; for example, in the USA the estimated prevalence was reported to be 19 in 10,000 children in 1992 increasing to 1 in 150 in 2002, 1 in 110 in 2006, and 1 in 88 in 2008 (Rice, 2012). ASDs are recognized as a common disorder today, with a median worldwide prevalence of 0.62% (Elsabbagh et al., 2012), and boys are affected by ASDs four times more frequently than girls. The increased prevalence of ASDs is most likely because of broadened diagnostic criteria and heightened awareness, but may also partially reflect a true increase due to environmental factors acting upon a genetically vulnerable background (King and Bearman, 2009; Lintas and Persico, 2009; Li et al., 2012).

In addition to a variable severity of the core deficits, ASDs patients also present other psychiatric and medical conditions, such as intellectual disability, epilepsy, motor control problems, attention-deficit/hyperactivity disorder, tics, anxiety, sleep disorders, and gastrointestinal problems (Simonoff et al., 2008; Lai et al., 2014).

For the past several decades, ASDs have been recognized as a complex brain disorder with high heritability, except with rare pedigrees, usually accompanied with other neurodevelopmental conditions shown to have Mendelian inheritance (Morrow et al., 2008; Novarino et al., 2012). Recent genomic and genetic studies have found that hundreds of genetic variants, including common and rare variants, contribute to the occurrence of ASDs. Rare and *de novo* mutations may pose a substantial risk for ASDs and play a substantial role in population risk, and common polymorphisms also contribute to ASDs. The role of individual alleles remains elusive and underestimated due to their small effect sizes (Murdoch and State, 2013). Many genes associated with ASDs play roles in the development and function of synapses, such as neuroligin 3 (*NLGN3*), *NLGN4X*, neurexin 1 (*NRXN1*), and SH3, and multiple ankyrin repeat domains 3 (*SHANK3*).

Post-mortem studies of ASDs patients have shown a reduction in the number of neurons in the amygdala, fusiform gyrus, and cerebellum and signs of persistent neuroinflammation (Lai et al., 2014). In addition, reduction in the density of serotonin transporters (5-HTT) was also found in the deep layers of the fusiform gyrus in autistic subjects (Oblak et al., 2013). Transcriptome analyses showed that genes involved in synaptic function were downregulated in the ASDs post-mortem brain. Moreover, the emergence of various types of genetically modified mouse models targeting ASDs-associated genes or loci in recent years have provided insights into particular aspects of ASDs. Therefore, it may be proposed that ASDs are a synaptic defect disease. In this review, we will focus on the role of synaptic-related genes in ASDs.

## SYNAPTIC PROTEINS, RECEPTORS, AND AUTISM SPECTRUM DISORDERS

### SYNAPTIC PROTEINS AND AUTISM SPECTRUM DISORDERS

#### Neurexin (NRXN)

Neurexins (*NRXN*) are a family of synaptic adhesion proteins that are located on the presynaptic membrane and bind to their postsynaptic counterpart, *NLGN*s. The *NRXN* family consists of three genes (*NRXN1*, *NRXN2*, and *NRXN3*), each of them generating a long mRNA encoding α-*NRXN* and a short mRNA encoding β-*NRXN* from two independent promoters. The intracellular domains of α-*NRXN*s and β-*NRXN*s are identical, whereas the extracellular domains are different. Specifically, the extracellular domains of α-*NRXN*s contain six laminin, nectin, and sex-hormone binding globulin (LNS) domains and three epidermal growth factor (EGF) domains, which form three repeated LNS (A)-EGF-LNS (B) structure. However, β-*NRXN*s have no EGF domain and only one LNS domain (Tabuchi and Sudhof, 2002). *NRXN*1, *NRXN*2, and *NRXN*3 are located on chromosomes 2p16.3, 11q13, and 14q31, respectively. α-*NRXN* triple knockout mice had reduced synaptic Ca^2+^ channel function, which causes impaired spontaneous and evoked neurotransmitter release (Missler et al., 2003).

Rare copy number variations and/or point mutations in *NRXN* genes have been repeatedly reported to be associated with ASDs (**Table 1**). Friedman and Luiselli (2008) first reported a 320 kb *de novo* heterozygous deletion of the *NRXN*-1α promoter and exons 1–5 in a boy with cognitive impairment, autistic features and physical dysmorphism. Later, the Autism Genome Project Consortium et al. (2007) identified a *de novo* heterozygous deletion that eliminated several *NRXN1* exons, including 1α and 1β, in two affected female siblings in one ASDs family. Several other studies have also reported deletions in *NRXN1* in ASDs patients (Kim et al., 2008; Marshall et al., 2008; Morrow et al., 2008; Glessner et al., 2009; Pinto et al., 2010; Bremer et al., 2011; Levy et al., 2011). To date, no homozygous deletions in *NRXN1* have been found, which may suggest that the dosage of *NRXN*1 is very important for neurological development. P300P, an *NRXN*1 common variant, was associated with ASDs in a Chinese ASDs patient. In addition to being associated with ASDs, *NRXN1* deletions have also been reported in other psychiatric conditions, such as schizophrenia, bipolar disorder, attention deficit hyperactivity disorder, and Tourette syndrome (International Schizophrenia Consortium, 2008; Walsh et al., 2008; Guilmatre et al., 2009; Zhang et al., 2009; Sundaram et al., 2010; Lionel et al., 2011).

**Table 1:** Summary of different defects in gene encoding for synaptic proteins in autism spectrum disorders.

Synaptic gene	Loci	Type of genetic defects	Reference
*NRXN1*	2p16.3	*de novo* heterozygous deletion	Autism Genome Project Consortium et al. (2007)
		Deletion	Pinto et al. (2010), Bremer et al. (2011)
* NRXN2*	11q13	Truncated mutation	Gauthier et al. (2011)
*NRXN3*	14q31	*de novo* deletion	Vaags et al. (2012)
		Inherited deletion	Vaags et al. (2012)
*CNTNAP2*		Homozygous mutation	Strauss et al. (2006)
		Rare non-synonymous variants	Bakkaloglu et al. (2008)
		Common polymorphisms	Alarcón et al. (2008), Sampath et al. (2013)
*NLGN1*	3q26	Common variants (rs1488545)	Talebizadeh et al. (2004), Gauthier et al. (2005)
*NLGN3*	Xq13	R451C transition	Jamain et al. (2003), Földy et al. (2013)
		Common variants (DXS7132)	Talebizadeh et al. (2004)
*NLGN4*	Xp22.3	Frameshift mutation (1186insT)	Laumonnier et al. (2004)
		Missense mutation	Yan et al. (2005)
		Deletion (1253delAG)	Laumonnier et al. (2004)
		Common variants (DXS996)	Talebizadeh et al. (2004)
*SHANK3*	22q13.3	Deletion	Durand et al. (2007), Gauthier et al. (2009)
		Novel non-synonymous variants	Moessner et al. (2007)
		Missense mutation	Gauthier et al. (2009)
		*de novo* heterozygous insertion	Durand et al. (2007)
*SHANK2*		*de novo* nonsense mutation	Berkel et al. (2010), Pinto et al. (2010)
*SHANK1*		Inherited deletion	Sato et al. (2012), Guilmatre et al. (2013)
*PSD-95*		Deletion	Feyder et al. (2010)
*Synapsin1*	Xp11.23	Nonsense mutation (Q555X )	Fassio et al. (2011)
		Mutations (A51G, A550T, T567A)	Fassio et al. (2011)
*Synapsin2*	3p25.2	Nonsense mutation (p.A94fs199X)	Corradi et al. (2014)
		Missense mutation (p.Y236S, p.G464R)	Corradi et al. (2014)
*Gephyrin*		Hemizygous deletions	Lionel et al. (2013)
		*de novo* deletion	Lionel et al. (2013)
		Paternally inherited deletion	Lionel et al. (2013)
*CDH13*	16q23	Recurrent larger genomic deletions	Sanders et al. (2011)
*PCDHA*		SNP	Anitha et al. (2013)
*PCDH9*		CNV	Betancur et al. (2009)
*PCDH10*		Homozygous deletion	Betancur et al. (2009)
*TAOK2*	16p11.2	Novel, recurrent microdeletion	Weiss et al. (2008)
		*de novo* deletion	Weiss et al. (2008)
		Reciprocal microduplication	Weiss et al. (2008)
*CNTN4*		Disruption	Roohi et al. (2009), Guo et al. (2012)

**NRXN*: neurexin; *CNTNAP2*: contactin associated protein-2; *NLGN*: neuroligin; *CDH*: cadherin; *PCDH*: protocadherin; *TAOK2*: thousand-and-one-amino acid 2 kinase; *CNTN*: contactin; CNV: copy number variation; SNP: Single-nucleotide polymorphism.*

A truncated mutation of *NRXN2* inherited from a father with severe language delay and a family history of schizophrenia was identified by Gauthier et al in an ASDs patient (Gauthier et al., 2011). *NRXN3* deletions have also been found in four ASD individuals: one was a *de novo* mutation, two were inherited from a non-affected mother or father, and one was inherited from a father with subclinical autism (Vaags et al., 2012).

*NRXN* animal models have provided evidence supporting the role of *NRXN* in ASDs pathology. *NRXN*1α KO mice showed a defect in excitatory synaptic strength, with a decrease in miniature excitatory postsynaptic current frequency and in the input–output relation of evoked postsynaptic potentials (Etherton et al., 2009). Behavioral studies have shown that *NRXN*-1 deficient mice display decreased prepulse inhibition and increased grooming behaviors but no obvious changes in social behaviors or spatial learning (Etherton et al., 2009). Studies in an α-*NRXN* triple KO mice with all three α-*NRXN*s (Nrxn1α/2α/3α) deleted have shown that α-*NRXN*s were not required for synapse formation but were essential for Ca^2+^-triggered neurotransmitter release (Missler et al., 2003).

Contactin associated protein-2 (CNTNAP2, also known as Caspr2) is a member of the *NRXN* superfamily and is involved in neuron–glia interactions and clustering K^+^ channels in myelinated axons. Strauss et al. (2006) identified a homozygous mutation of *CNTNAP2* in Amish children with PDD, seizures, and language regression (Strauss et al., 2006; **Table 1**). Bakkaloglu et al. (2008) found 13 rare non-synonymous variants unique to ASDs patients, which suggests that ASDs patients carry more *CNTNAP2* rare variants. Several other studies have also found other common polymorphisms of *CNTNAP2* that are associated with ASDs (Alarcón et al., 2008; Arking et al., 2008; Sampath et al., 2013). Interestingly, Alarcón et al. (2008) found that *CNTNAP2* provided a strong male affection bias in ASDs.

*CNTNAP2* KO mice exhibited deficits in the core ASDs behavioral domains, such as stereotypic motor movements, behavioral inflexibility, communication, and social behavior abnormalities (Peñagarikano et al., 2011).

#### Neuroligin (NLGN)

**FIGURE 1:**
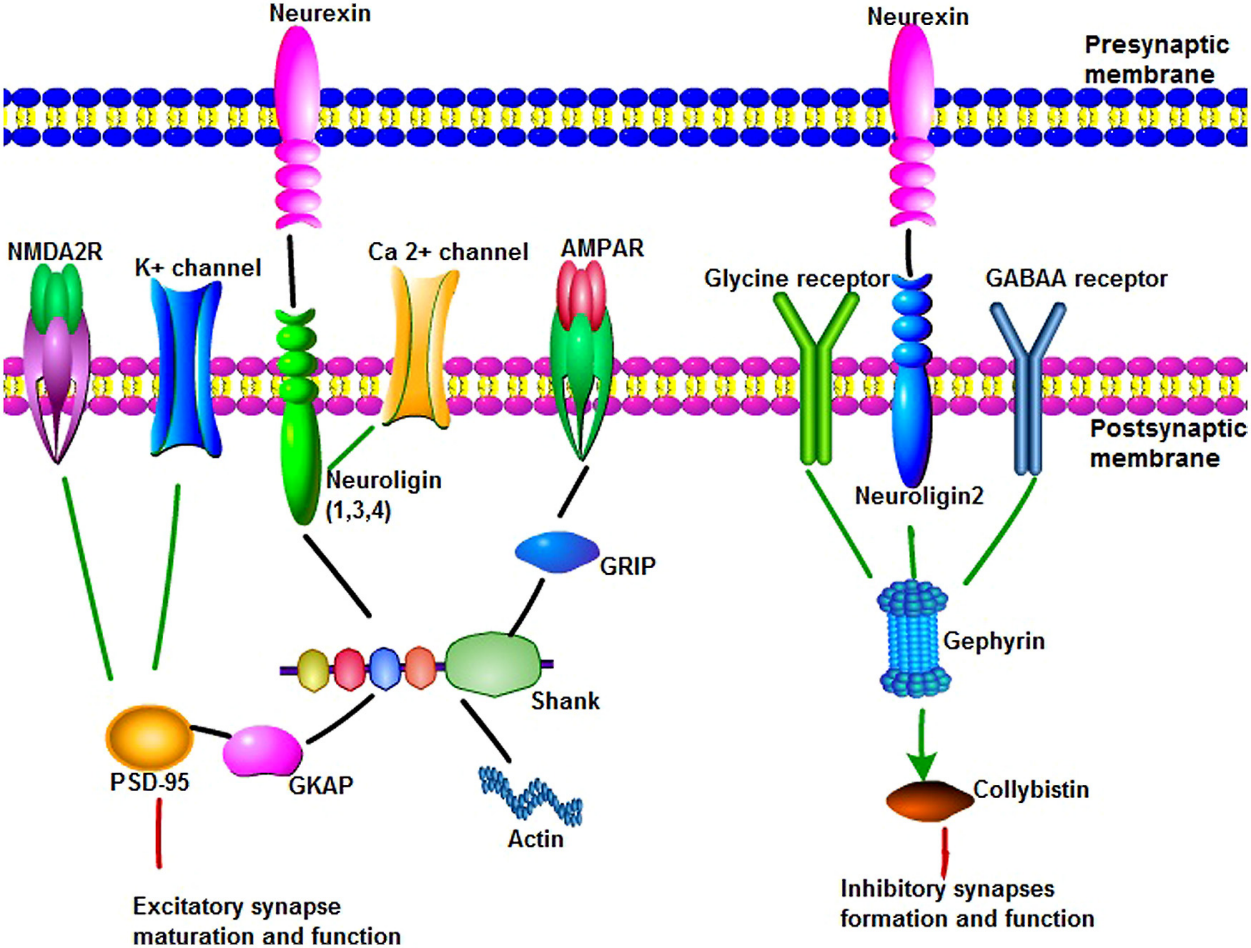
**The main synaptic proteins and receptors involved in this review in synaptogenesis and function.** (1) Neuroligins(*NLGN*s) can bind to neurexins (*NRXN*s), Ca^2+^ channel and shank through their different domains to play an important role in synapse maturation and function. *NLGN*s 1, 3, and 4 localize to excitatory synaptic sites, whereas *NLGN*-2 localizes primarily to inhibitory synapses. Shank are synaptic scaffold proteins that bind *NLGN*–*NRXN* and NMDA receptor complexes at the postsynaptic density of excitatory glutamatergic synapses. Multiple domains of shank are useful for protein–protein interaction, such as linking to actin cytoskeleton, binding to glutamate receptor-interacting protein to link with α-amino-3-hydroxy-5-methyl-4-isoxazolepropionic acid receptors. Postsynaptic density protein 95 (PSD-95) serves as a major functional bridge interconnecting the *NRXN*-*NLGN*-SHANK pathway. (2) Gephyrin is a key scaffold molecule of the postsynaptic membrane at inhibitory synapses. Gephyrin can interact with glycine receptor and alpha and beta subunits of the gamma-aminobutyric acid A (GABA_A_) receptor to mediate inhibition of synapse formation and function. *NLGN* 2 can bind to protein gephyrin and activate collybistin. *NLGN* 2, gephyrin, and collybistin complexes are sufficient to inhibitory neurotransmitter receptors clustering.

Neuroligins (*NLGN*) are a different type of synaptic cell adhesion proteins that are located in the postsynaptic membrane. *NLGN*s bind to their adhesive counterpart *NRXN*s and play an important role in synapse formation and function (**Figure 1**). The human *NLGN* family includes five *NLGN* genes (*NLGN*1, 2, 3, 4, 4Y), which are localized at 3q26 (*NLGN1*), 17p13 (*NLGN2*), Xq13 (*NLGN3*), Xp22.3 (*NLGN4*), and Yq11.2 (*NLGN4Y*). *NLGN*s contain a large extracellular domain that shares sequence homology with acetylcholinesterase and that is necessary for β-*NRXN* binding and synaptogenic activity, two EF-hand motifs that bind to Ca^2+^, an *O*-glycosylation region, a transmembrane domain, and a cytoplasmic C-terminal tail that contains a PSD-95/Dlg/ZO-1 (PDZ) interaction site (Dean and Dresbach, 2006; **Figure 1**). *NLGN*s-1, -3, and -4 localize mainly to glutamate synaptic sites, whereas *NLGN*-2 localizes primarily to gamma-aminobutyric acid (GABA) synapses (Missler et al., 2003; **Figure 1**). *In vitro* and* in vivo*, *NLGN*-1 overexpression increases excitatory synaptic responses and potentiates synaptic NMDA receptor (NMDAR)/AMPAR ratios. In contrast, *NLGN*-2 overexpression increases inhibitory synaptic responses. Accordingly, the inhibition of *NLGN*-1 expression selectively decreases the NMDAR/AMPAR ratio, whereas the deletion of *NLGN*-2 selectively decreases inhibitory synaptic responses. Furthermore, *NLGN*-1 expression selectively increases the maturation but not initiation of excitatory synapse formation in adult-born neurons (Chubykin et al., 2007; Schnell et al., 2012).

The earliest report regarding the potential association of *NLGN* genes and ASDs is Jamain et al. (2003; **Table 1**). In one ASDs multiplex family, the group found three ASDs siblings carrying one frameshift mutation (1186insT) of *NLGN4* that was inherited from the non-affected mother, creating a stop codon that led to a premature termination of the protein. In another family, a R451C transition in *NLGN3* that changed a highly conserved arginine residue into cysteine within the esterase domain was identified in two affected siblings. This point mutation was inherited from the non-affected mother. A study in a large French family found a 2 bp deletion (1253delAG) that resulted in a premature stop codon in the middle of the sequence of the normal *NLGN4* gene (Laumonnier et al., 2004). In this family, 10 males had non-specific X-linked mental retardation, two had autism, and one had pervasive developmental disorder. All affected patients had the same frameshift mutation. Missense changes in *NLGN4* were also found in Portuguese ASDs families (Yan et al., 2005). A study in a small Finnish autism sample did not find any functional mutation of *NLGN1*, *NLGN3*, *NLGN4*, or *NLNG4Y*, although three common variants (rs1488545 in *NLGN1*, DXS7132 in *NLGN3*, and DXS996 in *NLGN4*) that showed minor association with ASDs were found. Despite the evidence that *NLGN* is associated with ASDs, several studies have failed to find associations between rare mutations and common variants and ASDs (Talebizadeh et al., 2004; Vincent et al., 2004; Gauthier et al., 2005; Blasi et al., 2006; Wermter et al., 2008; Liu et al., 2013).

Several *NLGN* mutant mouse models have been developed to investigate the role of *NLGN* mutations in ASDs. Nlgn3 R451C knock-in mice, which corresponded to the human non-synonymous SNP (R451C) in *NLGN3* found in ASDs patients (Jamain et al., 2003), showed social interaction deficits and increased spatial memory and an electrophysiological phenotype consisting of increased inhibitory synaptic transmission in the somatosensory cortex (Tabuchi et al., 2007; Etherton et al., 2011a). Furthermore, researchers found that both *NLGN*-3 R451C-knockin and *NLGN*-3 knockout mutations in mice showed impairment in tonic endocannabinoid signaling (Földy et al., 2013). Another *NLGN3* mutation, R704C, was introduced into mouse *NLGN*-3 by homologous recombination, and electrophysiological and morphological studies have shown that although the *NLGN*-3 R704C mutation did not significantly alter synapse formation, it dramatically impaired synapse function. Moreover, the R704C mutation caused a major and selective decrease in AMPA receptor-mediated synaptic transmission in pyramidal neurons of the hippocampus, without similarly changing NMDA or GABA receptor-mediated synaptic transmission and without detectably altering presynaptic neurotransmitter release (Etherton et al., 2011b). Mice lacking the human *NLGN*4 (*NLGN*4-KOs) ortholog exhibited highly selective deficits in reciprocal social interactions and communication reminiscent of ASDs (Jamain et al., 2008).

#### Shank

Shank family proteins, also known as ProSAP, are synaptic scaffold proteins that bind *NLGN*-*NRXN* and NMDAR complexes at the postsynaptic density (PSD) of excitatory glutamatergic synapses (**Figure 1**). There are three genes that encode Shank proteins (*SHANK1*, *SHANK2*, and *SHANK3*). All Shank proteins are expressed in the brain but exhibit different patterns. Shank1 is expressed in most parts of the brain, except for the striatum, and it is highly expressed in the cortex and hippocampus. Shank2 and Shank3 are also present in the cortex and hippocampus. Shank2 is mostly absent from the thalamus and striatum, whereas Shank3 appears to be predominantly expressed in those regions. In the cerebellum, Shank2 is restricted to Purkinje cells, whereas Shank3 is restricted to granule cells (Sheng and Kim, 2000).* SHANK* directly or indirectly binds to* NLGNs* in the PSD. *In vitro* and* in vivo* studies highlight the important role of Shank3 for synaptic function. *SHANK3* functions as a scaffolding protein in spine morphogenesis and synaptic plasticity. Knockdown of *Shank3* in cultured hippocampal neurons leads to a reduced number and increased length of dendritic spines. When overexpressed in cultured hippocampal neurons,* Shank3* promotes the maturation and enlargement of dendritic spines (Betancur et al., 2009). Knock-down of *Shank3* in hippocampal neurons decreases spiny density, whereas transfection of *Shank3* in aspiny neurons induces the formation of dendritic spines with functional synapses (Betancur et al., 2009).

Shank contains multiple domains for protein–protein interaction, including ankyrin repeats (binding to α-fodrin to link to the actin cytoskeleton and calpain/calmodulin-mediated Ca^2+^ signaling), an SH3 domain (binding to glutamate receptor-interacting protein to link with α-amino-3-hydroxy-5-methyl-4-isoxazolepropionic acid (AMPA) receptors to the postsynaptic scaffold), a PDZ domain (binding different molecules within the PSD, including GKAP, to allow Shank to attach to PSD-95), a proline-rich region (which contains sites for Homer and cortactin), and a sterile alpha motif domain (which is involved in the polymerization of Shank molecules; Lim et al., 1999; Yoo et al., 2014; **Figure 1**).

*SHANK3* was the first gene in the *SHANK* family reported to be associated with ASDs (**Table 1**). The *SHANK3* gene is located on chromosome 22q13.3 within the critical region of 22q13.3 deletion syndrome (also known as Phelan-McDermid syndrome, PMS). 22q13.3 deletion syndrome is characterized by neonatal hypotonia, global developmental delay, absent or severely delayed speech, autistic behaviors, and intellectual disability (Phelan, 2008). The size of the deleted segments varied widely in individuals with this syndrome, but deletions of *SHANK3* were present in nearly all cases (Wilson et al., 2003, 2008; Dhar et al., 2010). Three ASDs families were observed to carry alterations of 22q and/or the *SHANK3* gene (Durand et al., 2007). In one family, an individual carried a *de novo* deletion of 22q13, in which the deletion breakpoint was located in intron 8 of SHANK3 and a 142 kb of the terminal 22q13 was removed. In the second family, two affected siblings were heterozygous for an insertion of a guanine nucleotide in exon 21, and the mutation was a *de novo* mutation. In the third family, a terminal 22q deletion was identified in a girl with autism who exhibited severe language delay. A 22qter partial trisomy in her brother who had Asperger syndrome was also identified, although the boy demonstrated precocious language development and fluent speech. These unbalanced cytogenetic abnormalities were inherited from a paternal translocation, t(14;22) (p11.2;q13.33). This finding suggests a gene dosage effect of *SHANK3*. A study of *SHANK3* variants by screening *SHANK*’s exon sequence in 400 ASDs families found 10 novel non-synonymous variants in ASDs (Moessner et al., 2007). Among these mutations, one was a *de novo* mutation, and the other nine were all inherited from one unaffected parent. Rare functional mutations of *SHANK3* have been identified in two ASDs families. One was a *de novo* deletion at an intronic donor splice site, and one was a missense mutation inherited from an epileptic father (Gauthier et al., 2009). Moreover, three deleterious variants (one 6-amino acid deletion upstream of the SH3 domain, one missense variant in the PDZ domain, and one insertion/deletion of a repeated 10 bp GC sequence located 9-bp downstream from the 3′ end of exon 11) were found in Japanese ASDs families (Waga et al., 2011). It has been reported that 2.3% of ASDs patients carry deleterious mutations in *SHANK3* (Boccuto et al., 2013).

Several studies have identified *de novo* deleterious mutations of *SHANK2* in ASDs (Berkel et al., 2010; Pinto et al., 2010; Leblond et al., 2012; **Table 1**). In one study, a *de novo* nonsense mutation, seven rare inherited changes and additional variants specific to ASDs were identified by sequencing *SHANK2* in 396 ASDs patients. In a separate study, *SHANK2* was sequenced in 455 patients. When combining the results of these two studies, a significant enrichment of variants affecting conserved amino acids in 29 of 851 (3.4%) patients and 16 of 1090 (1.5%) controls was observed. In neuronal cell cultures, the variants identified in patients were associated with reduced synaptic density at dendrites compared with the variants only detected in controls (Leblond et al., 2012).

The *SHANK1* gene rare mutation has also been associated with ASDs (Sato et al., 2012; **Table 1**). An inherited deletion of 63.8 kb encompassing *SHANK1* and the *CLEC11A* gene was found in a multigenerational family. In this family, four males carrying this deletion showed high-functioning autism or a broader autism phenotype, whereas the two females carrying the same deletion were not affected by ASDs (Sato et al., 2012). *SHANK1* deletions may be associated with high-functioning autism in males (Guilmatre et al., 2013).

Genetic mouse models resembling different *SHANK* mutations have been created to investigate the role of Shank in synapse conformation and function and its contribution to autistic pathology. Shank1 mutant mice (with a deletion of exons 14 and 15, which includes most of the PDZ region, resulted in the knockout of all detectable Shank1 protein in these animals) showed decreased movement in the open field and deficits in motor learning and contextual fear conditioning. Although these animals did not show apparent repetitive behaviors and seemed to have normal levels of social interaction, they showed a general deficit in social communicative behaviors by both ultrasonic vocalizations and urine-based communicative behaviors (Hung et al., 2008; Silverman et al., 2011; Wöhr et al., 2011). Shank2 mutant mice, mimicking the human microdeletion of exons 6 and 7, which targeted the PDZ domain and knocked out of all Shank2 isoforms, also showed alterations in behavior and synaptic plasticity (Schmeisser et al., 2012; Won et al., 2012). Shank3 exons 4–9 KO mice (resulting in the loss of the longest two isoforms of Shank3) were used to mimic human SHANK3 mutation by several research groups. Although the deficits in social interaction in these models were not consistent (Bozdagi et al., 2010; Peça et al., 2011; Yang et al., 2012), all of the models showed a repetitive self-grooming phenotype (Wang et al., 2011; Yang et al., 2012).

#### PSD-95

Postsynaptic density protein 95 (PSD-95; also known as DLG4, SAP90), is a member of the membrane-associated guanylate kinase family of synaptic molecules and serves as a major functional bridge interconnecting the *NRXN*-*NLGN*-SHANK pathway (**Figure 1**). *PSD-95* contains three PDZ domains, a single interior SH3 domain, and a COOH-terminal guanylate kinase domain. The cytoplasmic domains of all three *NLGNs* bind to the third PDZ domain of PSD-95, whereas NMDA2 receptors and K^+^channels bind to the first and second PDZ domains. *PSD-95* is localized at excitatory synapses and has been implicated in promoting synapse stability. *PSD-95* makes synaptic contacts more stable in older neurons than in younger neurons.

*PSD-95* knockout mice exhibit reduced AMPAR function and a decreased frequency of AMPAR-mediated miniature EPSCs, suggesting that* PSD-95* may regulate synaptic maturation through postsynaptic AMPA-type glutamate receptors (GluARs).

Thus far, no rare point mutation or CNV or common variants have been reported to be associated with ASDs, but *PSD-95* deletion (*Dlg*4^-/-^) mice have been shown to exhibit a complex range of behavioral and molecular abnormalities relevant to ASDs (**Table 1**). Dlg4^-/-^ mice showed increased repetitive behaviors, abnormal communication and social behaviors, impaired motor coordination, increased stress reactivity, and anxiety-related responses. *Dlg4*^-/-^ mice also had subtle dysmorphology of amygdala dendritic spines and altered forebrain expression of various synaptic genes (Feyder et al., 2010).

#### Synapsin

The synapsins are a family of presynaptic phosphoproteins that account for 9% of the vesicle protein and can regulateneurotransmitter release and neurite outgrowth (Rosahl et al., 1995). Synapsins contain a mosaic of conserved (A–C, E) and individual domains (D, F–J). They have three family members in mammals (*synapsin 1*,* synapsin 2*, and *synapsin 3*), which locate on chromosomes Xp11.23, 3p25.2, and 22q12.3, respectively. Cultured neurons from* synapsin 1,2,3*(–/–) triple knock-out mice exhibit severely dispersed synaptic vesicles and considerably reduced synaptic vesicles number (Fornasiero et al., 2012).

Mutations in* synapsin 1* (Q555X, A51G, A550T, and T567A) were found in a large French-Canadian family with epilepsy and ASDs (Fassio et al., 2011; **Table 1**). Furthermore, the nonsense Q555X mutation can reduce the phosphorylation caused by CaMKII and Mapk/Erk, which regulate synaptic vesicles trafficking and neurite outgrowth. The missense mutation of A550T and T567A can impair the targeting to nerve terminals (Fassio et al., 2011).

*Synapsin 2* has also been identified as an autism predisposing gene. In a study involving 190 individuals with ASDs, researchers found one nonsense mutation (p.A94fs199X) and two missense mutations of *synapsin 2* (p.Y236S and p.G464R; Corradi et al., 2014; **Table 1**).

*Synapsin* knockout mice may be identified as a useful experimental model of ASDs and epilepsy. Researchers found that *synapsin* knockout mice exhibit social novelty abnormality and avoidance behavior in social approach which are reminiscent of ASDs. Specifically, *synapsin 2* deletion mice display deficits in short-term social recognition and increased repetitive self-grooming behavior. *Synapsin 1* and *synapsin 3* deletion mice display an impaired social transmission of food preference. Synapsin 1and synapsin 2 deletion mice display a decreased environmental interest (Greco et al., 2013). The results demonstrate an involvement of synapsins in the development of the behavioral traits of ASDs.

#### Gephyrin

Gephyrin is a key scaffold molecule of the postsynaptic membrane at inhibitory synapses (**Figure 1**). It contains three domains, G domain in N-terminal, E-domain in C-terminal, and a large linker domain of the two. Gephyrin can interact with glycine receptor and alpha and beta subunits of the GABA_A_ receptor to mediate inhibition. *NLGN* 2 can bind to protein gephyrin through a conserved cytoplasmic motif and activate collybistin. *NLGN* 2, gephyrin, and collybistin complexes are sufficient to inhibitory neurotransmitter receptors clustering. Deletion of *NLGN* 2 in mice leads to a loss of recruitment of gephyrin at perisomatic but not dendritic sites (Poulopoulos et al., 2009; **Table 1**). Gephyrin-deficient mice die early postnatally and display loss of postsynaptic GABA(A) receptor and glycine receptors clustering, whereas glutamate receptor subunits were normally localized (Kneussel et al., 1999; Grosskreutz et al., 2003).

Exonic microdeletions in gephyrin gene have been reported a correlation with neurodevelopmental disorders including ASDs (Lionel et al., 2013). In one family, the proband with ASDs has a 357 kb *de novo* deletion in gephyrin and exhibits limited movement, slow motor development, and language delay. The second family has a 319 kb paternally inherited deletion in gephyrin and exhibits mild global developmental delay in early life, social difficulties, and repetitive behaviors. The third family has a 273 kb de novo deletion in gephyrin gene and exhibits developmental delay, cyclical seizures, and behavioral issues including anxiety, obsessive compulsive disorders, tics, and impulsive behaviors (Lionel et al., 2013).

#### Cadherins (CDHs) and protocadherins (PCDHs)

Cadherins (CDHs) are a family of glycosylated transmembrane proteins that mediate cell–cell adhesion, neuronal migration, spine morphology, synapse formation, and synaptic remodeling (Redies et al., 2012). Because the function of CDHs is dependent on the presence of Ca^2+^, they are named for the Ca^2+^-dependent cell adhesion molecule family. The CDH family is classified into classical CDHs, desmosomal cadherins, and protocadherins (PCDHs). Genome-wide association studies on a cohort of 4305 autistic subjects have shown that common variants between the *CDH9* and *CDH10* genes on chromosome 5p14.1 are associated with autism (Wang et al., 2009). Similarly, recurrent larger genomic deletions in 16q23 in *CDH13* was also observed in ASDs patients in 1124 ASDs families participating in genome-wide analyses (Sanders et al., 2011; **Table 1**). Furthermore, after the detection of 14 SNPs of protocadherin α in DNA samples of 3211 individuals with autism, 5 SNPs were showed significantly associated with autism (Anitha et al., 2013). In addition, CNVs in *PCDH9* and homozygous deletions in *PCDH10* have also been reported in ASDs (Betancur et al., 2009; **Table 1**).

*In situ* hybridization analysis in the embryonic and postnatal mouse demonstrated that CDH8 expression is restricted to specific developing gray matter structures. Later, a study using the PPL statistical framework identified that CDH8 is expressed in the developing human cortex of ASDs family, which implicates CDH8 in susceptibility to autism (Redies et al., 2012).

#### Thousand-and-one-amino acid 2 kinase (TAOK2)

Thousand-and-one-amino acid 2 kinase (TAOK2), also known as TAO2, is a serine/threonine-protein kinase that is encoded by the *TAOK2* gene in humans. It can activate mitogen-activated protein kinase (MAPK) pathways to regulate gene transcription. *TAOK2* interact with semaphorin 3A receptor neuropilin 1, which regulates basal dendrite arborization. In addition, *TAOK2* can be phosphorylated and activated by Sema3A. In cultured cortical neurons, *TAOK2* downregulation can decrease JNK phosphorylation and cause its inactivation. Furthermore, basal dendrite formation in cortical neurons caused by *TAOK2* downregulation can be rescued by active JNK1 overexpression. TAOK2 is involved in membrane blebbing, the DNA damage response, and the MAPK14/p38 MAPK stress-activated MAPK cascade. Recently, TAOK2 has been shown to play a role in basal dendrite formation (de Anda et al., 2012).

The *TAOK2* gene is located in the 16p11.2 chromosomal region. Approximately 1% of autistic subjects have been shown to have a novel, recurrent microdeletion, a *de novo* deletion of 593 kb on chromosome 16p11.2, and a reciprocal microduplication on chromosome 16p11.2 (Weiss et al., 2008), suggesting that 16p11.2 or the *TAOK2* gene is involved in susceptibility to ASDs (**Table 1**).

#### Contactin (CNTN)

Contactins (CNTNs) are members of the immunoglobulin superfamily. They are glycosylphosphatidylinositol-anchored neuronal membrane proteins and play important roles in axon growth and guidance and synapse formation and plasticity.

Array-based comparative genomic hybridization identified a paternally inherited chromosome 3 copy number variation in three autistic subjects. Specifically, a deletion in two siblings and a duplication in an unrelated individual were detected. Furthermore, these variations were mediated by disruptions of *CNTN4* (Roohi et al., 2009), suggesting that *CNTN4* may be involved in ASDs (**Table 1**). Recently, a study conducted in a Chinese population also came to the same conclusion (Guo et al., 2012). Although the sample sizes of these two studies were small, a CNV analysis involving 2195 autistic subjects indicated that CNTN4 deletions and duplications are associated with ASDs (Betancur et al., 2009). In a cohort of ASDs subjects, a CNV in the *CNTN5* gene was identified in one individual. In addition, a *CNTN6* deletion has also been found in an autistic family (Zuko et al., 2013).

Cntn6 knockout mice exhibited slower learning in terms of equilibrium and vestibular senses (Zuko et al., 2013), indicating that Cntn6-deficiency leads to defects in motor coordination. Other characteristics of ASDs, such as social interaction and social communication, remain to be determined in these mice. Cntn5 knockout mice exhibited decreased susceptibility to audiogenic seizures and impaired hearing, which may be related to the impairment of sensory information integration reminiscent of ASDs (Zuko et al., 2013).

### SYNAPTIC RECEPTORS AND AUTISM SPECTRUM DISORDERS

#### GABA receptors

Gamma-aminobutyric acid is the major inhibitory neurotransmitter in the human brain and is synthesized from excitatory neurotransmitter glutamate via the action of glutamate decarboxylase (GAD) enzymes, which have two main isoforms, GAD65 and GAD67. There are two main types of GABA receptors, ionotropic GABA_A_ receptors and metabotropic GABA_B_ receptors. GABA_B_ receptors are localized at pre-, post-, or extrasynaptic sites as functional heterodimers, whereas GABA_A_ receptors are the major mediators of fast inhibitory neurotransmission in the mammalian brain.

There are three GABA_A_ receptor genes (*GABRB3*,* GABRA5*, and* GABRG3*) localized on the human chromosome 15q11–q13, a part of the genome which is involved with genome instability, gene expression, imprinting and recombination and is one of the most complex regions in the genome (Martin et al., 2000). Duplications of the 15q11–13 locus have been observed in ASDs in several studies (Bolton et al., 2001; Kwasnicka-Crawford et al., 2007; Depienne et al., 2009). Duplication of the region containing GABA_A_ receptor subunits may lead to excessive inhibitory neurotransmission due to gene dosage; however, an *in vitro* study using a human neuronal cell line carrying a maternal 15q duplication showed that this variant leads to reduced GABRB3 expression via impaired homologous pairing (Meguro-Horike et al., 2011), suggesting that 15q11–q13 genes are regulated epigenetically at the level of both inter- and intra-chromosomal associations and that chromosome imbalance disrupts the epigenetic regulation of genes in 15q11–q13.

Moreover, mouse models mimicking human 15q11–q13 duplication have exhibited features of autism, such as poor social interaction, behavioral inflexibility, and abnormal ultrasonic vocalizations (Nakatani et al., 2009).

GABA_B_ receptors play an important role in maintaining excitatory-inhibitory balance in brain. In autistic brain subjects, researchers have found that the expression of GABA_B_ receptor subunits GABA_B_ receptor 1 (GABBR1) and GABA_B_ receptor 2 (GABBR2) were significantly reduced (Fatemi et al., 2009). Furthermore, clinical trials show that the selective GABA_B_ receptor agonist STX209 (arbaclofen) has a potential to improve social function and behavior in patients with fragile X syndrome and was generally well-tolerated in ASDs individuals (Berry-Kravis et al., 2012; Erickson et al., 2014).

*In vitro*, STX209 (arbaclofen, R-baclofen) can correct the elevated basal protein synthesis in the hippocampus of Fmr1-knockout mice, an animal model of Fragile X syndrome. *In vivo*, acute administration of STX209 can decrease mRNA translation in the cortex of Fmr1-knockout mice. Furthermore, the chronic administration of STX209 in juvenile mice can improve the increased spine density in Fmr1-knockout mice (Henderson et al., 2012). Since ASDs individuals have something in common with Fragile X syndrome, this implies that GABA_B_ receptor agonist STX209 may also improve synaptic abnormalities in ASDs.

Consistent with the genetic evidence for the involvement of GABAergic genes in ASDs, the expression of GABAergic genes and related proteins have been reported to be reduced in the post-mortem ASDs brain. GAD65 and GAD67 proteins were reduced in the cerebellum and parietal cortex (Fatemi et al., 2002), GAD67 mRNA was reduced in cerebellar Purkinje cells (Yip et al., 2007), and GABA_A_ receptor binding was reduced in the hippocampus (Blatt et al., 2001) and anterior and posterior cingulate cortices (Oblak et al., 2009, 2011).

Although technical difficulties still exist, researchers have attempted to measure GABA function *in vivo*, and these results support the presence of GABAergic defects in ASDs patients. Using proton magnetic resonance spectroscopy ([^1^H]MRS; Harada et al., 2011) reported that GABA concentrations were reduced in the frontal cortex of ASDs children, whereas no differences were observed in the basal ganglia (Harada et al., 2011). Two studies using SPECT (Single Photon Emission Computed Tomography) found reductions in GABA_A_ receptors in both ASDs adults and children (Mori et al., 2012; Mendez et al., 2013).

In addition to the genes/proteins involved directly in GABA synthesis and transmission, many other factors exert indirect effects on GABA functioning through the regulation of gene expression, receptor trafficking, and downstream signaling pathways; therefore, GABAergic dysfunction could also be a downstream consequence of mutations in the genes involved in the increase or decrease of GABA transmission. CNTNAP2, which is a part of the *NRXN* family, has been associated with autism (Gregor et al., 2011; Stein et al., 2011). CNTNAP2 knockout mice showed specific deficits in inhibitory signaling, with reduced GAD1 expression and a reduced number of GABAergic interneurons (Pe Peñagarikano et al., 2011). Similar findings have been observed in another ASDs candidate gene model; CADPS2 knockout mice showed reduced cortical parvalbumin GABA interneurons and a reduced number of cerebellar Purkinje cells (Sadakata et al., 2007).

#### Glutamate receptors

Glutamate is the major excitatory neurotransmitter in the human brain. Glutamate receptors (GluARs) are composed of ionotropic glutamate receptors (iGluRs) and metabotropic glutamate receptors (mGluRs). Findings from genetic studies, post-mortem brain studies, animal models, and clinical drug trials have implicated a dysfunctional glutamatergic system in ASDs; however, hypo- and hyperfunction coexists in different forms of ASDs.

Ionotropic glutamate receptors are classified into NMDA (*N*-methyl-D-aspartate), AMPA (2-amino-3-hydroxy-5-methyl-4-isoxazolepropionic acid), and kainate receptors based on structural, pharmacological, and physiological properties. iGluRs are tetramers encoded by 18 genes. NMDARs are obligate heteromers formed as tetramers from the co-assembly of GluN1, GluN2A-GluN2D, GluN3A, and GluN3B subunits. Each NMDAR channel contains a combination of two GluN1 and two GluN2A–GluN2D subunits or two GluN1 with one GluN2 and one GluN3 subunit. AMPARs are homo- or hetero-tetramers formed from the GluA1–GluA4 subunits and are Mg^2+^-insensitive. Kainate receptors are tetramers formed from combinations of the GluK1–GluK5 subunits.

Several genetic studies have reported that *NMDARs* genes are associated with ASDs. Two studies that sequenced ASDs patients identified rare disruptive mutations in the GluN2B (*GRIN2B*) gene (Tarabeux et al., 2011; O’Roak et al., 2012). Common polymorphisms in *GRIN2B* and *GRIN2A* have also been associated with ASDs (Barnby et al., 2005; Yoo et al., 2012). Interestingly, the NMDAR subunits have differential expression during development, with GluN2B expressed early in development, followed by GluN2A during later development and synapse maturation (Sanz-Clemente et al., 2013).

Ramanathan et al. (2004) identified a 19 mb deletion of chromosome 4q in an ASDs child, which included the AMPA 2 gene that encodes the glutamate receptor GluR2 sub-unit (Ramanathan et al., 2004). One study identified chromosome 6q21 as a candidate region for autism and found a functional SNP in glutamate receptor 6 (*GluR6* or *GRIK2*) gene associated with ASDs (Jamain et al., 2002).

A post-mortem brain study also found that ASDs patients have specific abnormalities in AMPA receptors and glutamate transporters in the cerebellum (Purcell et al., 2001). The mRNA levels of excitatory amino acid transporter 1 and glutamate receptor AMPA1 (GluA1) were significantly increased in autism subjects, and AMPAR density was decreased in the ASDs cerebellum (Purcell et al., 2001).

Parvalbumin-selective NMDAR 1 knockout (NR1 KO) mice exhibited autism-like phenotypes compared with wild-type mice; the N1 ERP latency was delayed, sociability was reduced, and mating USVs were impaired (Saunders et al., 2013).

The administration of acute PCP and ketamine, NMDAR antagonists, has been shown to mimic the symptoms of autism in humans (Carlsson, 1998). Based on this phenomenon and neuroimaging and neuroanatomical studies, Carlsson (1998) proposed that infantile autism is a hypoglutamatergic disorder. Recently, both the use of an NMDAR agonist and antagonist has been reported in ASDs patients. Daily doses of D-cycloserine, an NMDAR glycine site partial agonist, significantly improved social withdrawal (Posey et al., 2004), and daily doses of amantadine (memantine), an NMDAR non-competitive antagonist, reduced some negative symptoms of autism, such as hyperactivity (King et al., 2001; Chez et al., 2007).

mGluRs are members of the group C family of G-protein-coupled receptors. mGluRs have seven transmembrane domains that span the cell membrane. Differently to iGluRs, they are not ion channels. There are eight different types of mGluRs, namely mGluR1 to mGluR8, which are divided into three groups, group 1, group 2, and group 3. mGluR1 and mGluR5 belong to group 1 family, mGluR2, mGluR3, and mGluR4 belong to group 2 family, and mGluR6, mGluR7, and mGluR8 belong to group 3 family. They can regulate neuronal excitability, learning, and memory.

A study using high-throughput multiplex sequencing revealed significant enrichment of rare functional variants in the mGluR pathway in non-syndromic autism cases. (Kelleher et al., 2012). Most recently, in a valproate-induced rat model of autism, the expressions of mGluR2/3 protein and mGluR2 mRNA were found significantly reduced. *N*-acetylcysteine (NAC) recued social interaction and anxiety-like behaviors of the VPA-exposed rats. In addition, these effects can be blocked by intra-amygdala infusion of mGluR2/3 antagonist LY341495 (Chen et al., 2014). These results indicate that the disruption of social interaction in VPA induced rats could be restored by NAC, which may depend on the activation of mGluR2/3.

A decrease in mGluR has been found in PTEN knockout mice showing autism-like behavioral deficits (Lugo et al., 2014). By reducing 50% of mGluR5 expression, several abnormalities of Fmr1 knockout mice can be rescued. For example, density of dendritic spines on cortical pyramidal neurons and basal protein synthesis in hippocampus are increased, inhibitory avoidance extinction and audiogenic seizures are improved (Dolen et al., 2007).

## CONCLUDING REMARKS

In this review, we have summarized findings about some synapse proteins and receptors linked to ASDs. Due to different sample sizes and research methods, some results need further replication in additional and larger samples. For some of the synapse protein defects described in this review, animal model studies are lacking. Furthermore, genetic mutations only have been found in some ASDs subjects. Many patients do not exhibit these types of changes. Other signaling pathways, such as MAPK/JNK, have been correlated with synapse pathways in the pathogenesis of ASDs. Therefore, an intriguing question for future work is whether other signaling pathways have crosstalk with synapse pathways during the occurrence of ASDs.

## Conflict of Interest Statement

The authors declare that the research was conducted in the absence of any commercial or financial relationships that could be construed as a potential conflict of interest.
